# Oral dosing of rodents using a palatable tablet

**DOI:** 10.1007/s00213-018-4863-2

**Published:** 2018-03-06

**Authors:** Sandeep S. Dhawan, Shuang Xia, David S. Tait, Christoffer Bundgaard, Ellen Bowman, Verity J. Brown

**Affiliations:** 1 School of Psychology and Neuroscience, University of St Andrews, St Mary’s Quad, South Street, St Andrews, Fife, KY16 9JP UK; 2 H. Lundbeck A/S, Discovery DMPK, 9 Ottiliavej, DK-2500 Valby, Denmark

**Keywords:** Modafinil, Oral administration, Pharmacokinetics, Locomotor activity

## Abstract

**Rationale:**

Delivering orally bioavailable drugs to rodents is an important component to investigating that route of administration in novel treatments for humans. However, the traditional method of oral gavage requires training, is stressful, and can induce oesophageal damage in rodents.

**Objectives:**

To demonstrate a novel administrative technique—palatable gelatine tablets—as a stress-free route of oral delivery.

**Methods:**

Twenty-four male Lister hooded rats were sacrificed for brain tissue analysis at varying time-points after jelly administration of 30 mg/kg of the wake-promoting drug modafinil. A second group of 22 female rats were tested on locomotor activity after 30 mg/kg modafinil, or after vehicle jellies, with the locomotor data compared to the brain tissue concentrations at the corresponding times.

**Results:**

Modafinil was present in the brain tissue at all time-points, reducing in concentration over time. The pattern of brain tissue modafinil concentration is comparable to previously reported results following oral gavage. Modafinil-treated rats were more active than control rats, with greater activity during the later time-periods—similar to that previously reported following intraperitoneal injection of 40 mg/kg modafinil.

**Conclusions:**

Palatable jelly tablets are an effective route of administration of thermally stable orally bioavailable compounds, eliminating the stress/discomfort and health risk of oral gavage and presenting as an alternative to previously reported palatable routes of administration where high protein and fat levels may adversely affect appetite for food reward, and uptake rate in the gastrointestinal tract.

## Introduction

Administration of pharmacological agents in experimental animals, to investigate effects on the brain and behaviour, is performed via a variety of routes—e.g. intra-cerebrally (via implanted catheters in the brain); by injection (intraperitoneal—i.p.; subcutaneous—s.c.; intravenous—i.v.; or intramuscular—i.m.); transdermally (via a skin-patch; or onto a mucous membrane); by inhalation; orally (by gavage—insertion directly into the stomach; or through mixing with food or drink). The route chosen will impact the pharmacokinetics (e.g. absorption rate), and therefore influence the timing and magnitude of any behavioural effect, but there are also other practical considerations when selecting a particular route of administration. For example, there may be secondary behavioural effects of giving the drug: an interruption of ongoing behaviour might distract or arouse the animal, such that behaviour after drug administration changes irrespective of any pharmacological effect of the drug. This is likely to be a particular problem if the route of administration causes pain or discomfort, as is evident with needle-sticks and gavage. Oral gavage has been reported to induce significant increases in heart rate 2–5 h post-gavage and increase faecal corticosterone (Walker et al. 2012; Bonnichsen et al. 2005); and stress-related arousal will have behavioural consequences. Yet oral administration is a desirable route to explore, given the ultimate preference for such in treatments for human conditions, so establishing stress-free oral route for laboratory animals is a priority.

Walker et al. (2012) have shown that mice which voluntarily consumed a ‘pill’ made from Transgenic Dough Diet™ (Bioserve, Inc.) did not show a stress response compared to oral gavage. Whilst it is simple to knead drugs into the dough, there are disadvantages to using this diet: it is designed for rodents with chewing, dental or mobility impairments and therefore whilst highly palatable, is also high in protein and fat. This makes it less useful for studies that measure behaviour motivated by food and in instances where uptake may be affected by food in the gastrointestinal tract. Other low-stress palatable techniques, such as adding drugs to condensed milk (Murphy et al. 2015), are similarly disadvantaged by high protein, fat and sugar content; whilst training rats to drink from a syringe (e.g. Mar et al. 2017; Robinson 2012) requires a time component for both training and actual experimental dosing.

In the present study, we used a flavoured, but fat-/sugar-free, gelatine “jelly” tablet (previously described in Bowman et al. 2014) to orally administer modafinil (2-[(Diphenylmethyl)sulfinyl]acetamide) to rats. Modafinil is a stimulant drug used in the treatment of narcolepsy and excessive sleepiness (Bastuji and Jouvet 1988; Edgar and Seidel 1997). It has gained particular interest for its unique wake-promoting effects without exerting typical amphetamine-like side-effects, such as sleep rebound, and neither does it have abuse potential (Edgar and Seidel 1997; Deroche-Gamonet et al. 2002; Touret et al. 1995; Leith and Barrett 1976; Koob and Bloom 1988).

Modafinil has also been investigated for its potential cognitive-enhancing effects, where it has been linked to increased performance and accuracy on a variety of cognitive tasks in both patients with schizophrenia and healthy adults (digit span task, CANTAB-Stroop), as well as in experimental animals performing visual attentional tasks and T-maze-serial reversal learning (Minzenberg and Carter 2008; Turner et al. 2003; Randall et al. 2005; Morgan et al. 2007; Beracochea et al. 2002). We have recently observed effects of 30 mg/kg modafinil, administered i.p. prior to testing, on Lister hooded rat behavioural flexibility during the intradimensional/extradimensional (ID/ED) attentional set-shifting task (Chase, Tait and Brown, unpublished observations).

To determine the viability of the oral jelly method of administration, we investigated the effects of a single dose of modafinil on locomotor activity (LMA) and brain tissue concentration. Previous studies have shown that various doses of modafinil in the rat elicit either an outright increase in LMA (75–600 mg/kg; Ishizuka et al. 2008; Rowley et al. 2014), or a slowed reduction (40 mg/kg; Simon et al. 1996), compared to controls. Using an electroencephalogram (EEG), Edgar and Seidel (1997) observed that their (100–300 mg/kg) modafinil-induced LMA ‘increase’ derived from time spent awake—i.e. that “LMA intensity” (LMA per time spent awake) did not change. Based on our prior behavioural observations after 30 mg/kg i.p., the robust effect of 40 mg/kg i.p. on LMA (Simon et al. 1996), and an established pharmacokinetic profile after 32 mg/kg by oral gavage (Waters et al. 2005), we investigated oral jelly administration of 30 mg/kg modafinil—predicting it would induce a similar LMA profile to that observed by Simon et al. (1996): a reduction in LMA over time that was slower than that observed in controls. We also explored modafinil concentrations in brain tissue to establish a pharmacokinetic profile for the oral jelly administration route after the same dose, to allow comparison to the oral gavage route.

## Methods

### Animals

Forty-six (24 male; 22 female) naïve Lister hooded rats (Charles River, UK) were group-housed, but segregated by sex, and maintained on a 12 h light/dark schedule (lights-on at 7 am). They were maintained on a diet of 15–20 g of standard laboratory chow each day with water available ad libitum. The male rats weighed between 480 and 630 g and the females weighed between 185 and 250 g over the course of the experiment. All experimental procedures were carried out in accordance with the UK Animals (Scientific Procedures) Act 1986 and EU Directive 2010/63/EU.

### Drug preparation and habituation

Modafinil was administered to the rats orally, suspended in a palatable gelatine (jelly) tablet as the vehicle. The jellies were made by heating a water bath to ~ 70 °C, then placing into the water bath a beaker containing 50 ml of flavoured, sugar-free, fruit juice concentrate (Robinsons Squash, Britvic PLC, UK) and adding 12 g gelatine (Dr. Oetker, UK). The mixture was stirred until the gelatine was fully dissolved. Modafinil (Sequoia Research Products Ltd., UK) doses (30 mg/kg) for individual rats were weighed out and added to the bottom of 2 ml wells in a plastic mould. The gelatine solution was then pipetted into the wells (1.5 ml/well), and the mixture carefully stirred with a small pipette tip to suspend the modafinil. Vehicle jellies were made using the same procedure, but without modafinil. The plastic mould was then placed in a fridge (3–5 °C) overnight for the jellies to cool and set. Once the jellies were set, they were removed from the moulds and stored in the fridge in airtight containers.

The rats were habituated to vehicle jellies before data collection: rats were placed individually in a large home-cage and presented with a jelly in a small ceramic pet food bowl, and left until they had fully consumed it. This was repeated once per day until rats were eating the jelly within 5 min, which was usually by the third day.

### Experiment 1: the pharmacokinetic profile of orally administered modafinil

#### Drug administration

On the day of the experiment, the 24 male rats were single-housed and presented with modafinil-containing jellies. The time at which a rat finished eating the jelly was recorded (typically no more than 5 min after it had started eating), and at specific time-points after that (15, 30, 45, 60, 75, 90, 120 and 150 min; *n* = 3 per time-point) rats were sacrificed by decapitation. After decapitation, brains were extracted from the skull, the cerebellum was removed, and then the remainder was bisected in the sagittal plane. Each hemisphere was weighed and then rapidly frozen by immersion in isopentane (Sigma-Aldrich, UK) chilled by dry ice. The hemispheres were then wrapped in aluminium foil, individually placed in homogenisation tubes and stored at − 80 °C.

#### Post-mortem bioanalysis

Rat brain concentrations of modafinil were determined using ultra performance liquid chromatography (UPLC) coupled to tandem mass spectrometry (MS/MS). Brain samples were prepared by homogenising the brains 1:3 (*v*/*v*) with a mixture of water, 2-propanol and dimethyl sulfoxide (DMSO; 50:30:20 *v*/*v*/*v*). Samples were precipitated with acetonitrile and DMSO (80:20 *v*/*v*) containing internal standard. Following centrifugation, 10 μl was injected onto the chromatographic system consisting of an Aria TLX2 system (Thermo Fisher Scientific Inc., MA, USA) connected to a Thermo TSQ Quantum Ultra triple quadrupole mass spectrometer. Analytical separation was achieved using a Kinetex C18 column (50 × 2.1 mm, 2.6 μm particles; Phenomenex, CA, USA). The mobile phase consisted of 0.01% formic acid in acetonitrile and 0.1% formic acid in water pumped through the column using a 3-min gradient. Modafinil was detected at a parent > daughter mass to charge ratio (*m*/*z*) of 274.01 > 167.00. The retention time was 1.27 min. The peak area correlated linearly with the brain concentration of the analyte in the range of 40–4000 ng/g brain.

### Experiment 2: the effects of orally administered modafinil on locomotion activity

#### Test procedure

On the day of testing, starting 4 h after lights on, the 22 female rats were habituated to a 7 × 15 LED infrared actimeter (Hamilton-Kinder *MotorMonitor*; Ponway, CA, USA) for 60 min. Following actimeter habituation, a jelly was presented in a ceramic bowl directly into the actimeter. Jellies were administered 5 h after lights-on (Circadian time 5 (CT5), where CT0 is lights-on), as it has been observed that sleep loss can impact stimulant drug efficacy (Edgar et al., 1991; Roehrs et al., 1989). CT5 (in 12 h light/dark schedule) in particular, yields the least interference from the high variability in wakefulness due to large amounts of sleep (< CT5) and the normal circadian wakefulness found closer to lights-off (> CT5) (Edgar & Seidel, 1997). Precisely 15 min from the point the rat started eating the jelly (with all jellies consumed within 5 min), the actimeter was reset, and testing commenced. LMA data were compiled to investigate time-periods of interest (15–30, 30–45, 45–60, 60–75, 75–90, 90–105, 105–120, 120–135, 135–150 and 150–160 min). Timings were aligned so that sacrifice time-point ‘15 min’ from experiment 1 falls within the first 5 min of the ‘15–30 min’ time-period from experiment 2 (and so on for the remaining time-points/periods—with the last two sacrifice time-points from experiment 1 falling within the last four time-periods of experiment 2).

#### Counterbalancing

Each rat was tested twice, with 11 rats each receiving modafinil and vehicle jellies in each test, and rats receiving modafinil and vehicle jellies once each. There was a minimum of 5 days between tests to allow for washout of modafinil.

#### Data compilation and analysis

The LMA data were configured as LMA/min over 10 time-periods (15–30, 30–45, 45–60, 60–75, 75–90, 90–105, 105–120, 120–135, 135–150 and 150–160 min), and analysed by ANOVA using SPSS v. 22 with the dependant variable being the total number of infrared beams crossed within the observed time-period. There were two within-subjects factors: dose (two levels: modafinil and vehicle), and time-period (ten levels: as above). A second ANOVA, with two within-subjects factors: test (test 1 and test 2) and time-period was used to confirm that rats’ performance was not affected by test order. Greenhouse-Geisser corrections were applied for sphericity violations.

## Results

### Experiment 1: the pharmacokinetic profile of orally administered modafinil

**Fig. 1:**
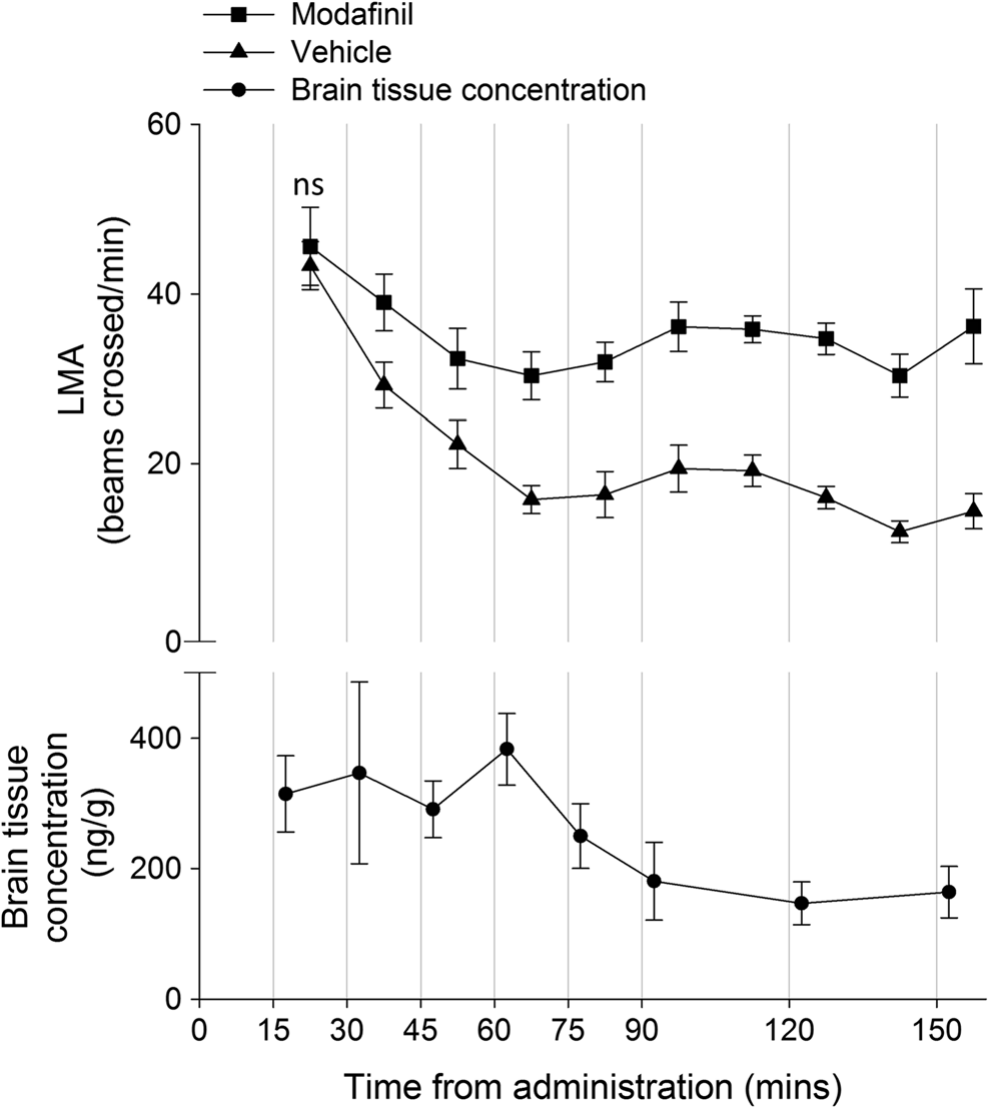
Locomotor activity: mean ± SEM number of infrared beams crossed after oral administration of either 30 mg/kg modafinil or vehicle jellies during time-periods from 15 to 160 min after administration. The wake-promoting effects of modafinil became obvious after the first time-period (“ns” denotes the single time-period where there was no significant difference between the groups), following a greater reduction in exploratory behaviour in the control group. Pharmacokinetic profile: mean ± SEM ng/g modafinil in brain tissue collected at specific time-points (within the first 5 mins of the 15 mins LMA time-periods) after oral administration of 30 mg/kg modafinil. Concentration reduced over time, although high variability at the 30-min time-point (within the 30–45-min time-period) may mask a true peak at the 60 min time-point (within the 60–75 min time-period)

Modafinil was detected in the brain tissue at all time-points (Fig. 1). A rapid uptake in the brain was observed with mean concentrations in the range of 300–400 ng/g during the first hour after drug intake, where after a gradual decrease in brain concentrations was observed over time.

### Experiment 2: the effects of orally administered modafinil on locomotion activity

There was no effect of running the test twice: as a group, the rats were equally active in both tests (main effect of test: *F* (1, 21) = 1.19, not significant (ns)) and the time course of activity was also similar in each test (test by time-period interaction: *F* (4.72, 99.18) = 1.63, ns).

Overall, modafinil administration resulted in greater LMA compared to vehicle-treated rats (main effect of dose: *F* (1, 21) = 49.19, *p* < 0.05; Fig. 1). Furthermore, as previously observed by Simon et al. (1996), whilst the LMA of control rats decreased rapidly, that of modafinil-treated rats was less reduced (main effect of time-period (*F* (4.35, 91.25) = 13.91, *p* < 0.05); dose by time-period interaction (*F* (6.73, 141.34) = 4.03, *p* < 0.05)). Specifically, Bonferroni-corrected pairwise comparisons showed that modafinil-treated rats were significantly more active in all time-periods other than the first compared to vehicle-treated rats: there was a distinct pattern of reducing activity in the vehicle-treated rats over the course of the first few time-periods (LMA is significantly lower in all time-periods after the first, and in most after the second), with LMA stabilising at the 60–75 min time-period; whereas the modafinil-treated rats’ LMA barely reduced, differing significantly only between the first and third time-periods.

## Discussion

We have investigated the efficacy of a novel route of administration, in the form of a suspension in a palatable jelly tablet, for thermally stable, bio-orally available drugs. We have shown that modafinil is present in the brain up to at least 150 min after consumption of a 30 mg/kg modafinil-containing jelly, and that LMA is affected by modafinil on a similar timescale. This delivery method was presented as a reduced-stress alternative to oral gavage—a forced oral route of administration, which elicits undesirable stress responses and is known to alter an animal’s response to pharmacological agents (Brown et al. 2000; Roberts et al. 1995). The results we obtained from the pharmacokinetic profile of modafinil concentration in brain tissue show a pattern comparable to previously published data from oral administration of 32 mg/kg modafinil via gavage (Waters et al. 2005). The concentration levels reported by Waters et al. are, however, substantially higher at the 30–60-min time-points than those reported here—although unlike the rats in our study, which were on our standard food control regime for food-motivated behavioural testing, their rats were deprived of food overnight prior to the administration of modafinil. Despite this seeming discrepancy, both our data and that of Waters et al. demonstrate a rapid decrease in concentration after the 60-min time-point. Whilst our data shows a gradual decrease in modafinil concentration as time progresses, it is clear from Fig. 1 that our data have high variability in the early time-points, with the greatest concentration mean at 60 min—the same as reported in Waters et al. Although no statistical analysis of Waters et al.’s data was presented, the standard deviation data suggest very high variability at their two highest concentrations—30 and 60 min. Both our study and that of Waters et al. sampled three rats per time-point, and given the variability in their data at the highest concentrations reported, we do not think it reasonable to conclude that there is a substantial difference between the pharmacokinetic results. Furthermore, unpublished data from oral gavage using a dose of 64 mg/kg show a concentration of 350 ng/g modafinil in brain tissue at 60-min post-administration, although again with high variability (Bundgaard, unpublished observations)—comparing favourably to our reported 383 ng/g concentration at the same time-point.

Our data also demonstrate that rats fed modafinil in jelly form show LMA that compares favourably to data at a similar dose after i.p. administration: Simon et al. (1996) report an effect on LMA after 40 mg/kg modafinil. As in that study, we have shown that following actimeter habituation, compared to control performance, modafinil-treated rats exhibited greater LMA overall and importantly, continuously after the first time-period. Both our data and that of Simon et al. illustrate a more rapid decline in LMA in vehicle-treated than modafinil-treated rats, followed by stabilisation during later time-periods. Our observations differ, however, in that after the first time-period, modafinil-treated rats are consistently more active than vehicle-treated—whereas Simon et al. report differences only at three time-periods: 10–20, 30–40 and 70–80 min. That we used more than the twice the number of subjects, and a within-subjects design, suggests that lower variability in our sample accounts for our more robust effect—rather than, for example, a gender or strain effect.

As previously reported by Edgar and Seidel (1997), EEG recordings support the conclusion that modafinil causes an increase in LMA only in proportion to the expected time spent awake, and that LMA intensity is not affected by modafinil. In contrast, amphetamine-like stimulants not only increase LMA intensity, but also result in stereotyped behaviours such as “compulsive licking, sniffing, biting, chewing, grooming and head-waving” (Duteil et al. 1990). Whilst 300 mg/kg doses of modafinil do not yield increased LMA intensity in rats (Edgar and Seidel 1997), 600 mg/kg is reported to result in “intense chewing and sniffing… interrupted by brief bursts of locomotor activity” (Rowley et al. 2014).

Whilst the aim of jelly administration is to present a stress-free alternative to gavage as a route of oral administration, we recognise that our data do not show the effects of any reduction in stress—given their similarity to published data where either i.p. or oral gavage administration routes were used. It is the case that many experiments would not be sensitive to the changes between stress-free oral administration and gavage/i.p. administration. However, we consider the obvious benefits of a non-aversive means of dosing an animal to manifest when using drugs with a short profile of activity—which might require multiple dosings during a single experiment. For example, we have found that rats become reluctant to engage with a task (ID/ED attentional set-shifting) once they have learned to associate the task with an i.p. injection—i.e. when we have need to administer via i.p. in the middle of a test, on subsequent tests, rats are more reluctant to participate, as the expectation of an injection affects their interest in the task (Tait and Brown, unpublished observations). Thus, whilst we may not observe the effects of stress-reduction on the actual data, the benefits for collecting those data are obvious.

The jelly administration method presents as an alternative to other oral routes: gavage, syringe-feeding, and the ‘pill’ method described in Walker et al. (2012). Rich palatability and a capacity to manufacture a higher volume of pills per batch, makes the Transgenic Dough Diet a viable alternative to oral gavage, but the high fat and protein content is less desirable for food-motivated experiments, and where there is likely to be slower absorption of a drug because of gastrointestinal contents. Additionally, the dough diet requires the drug to be kneaded in (Walker et al. 2012), and therefore final concentration may be inconsistent as there may be irregular distribution within the dough unless pills are made individually. Some drugs also require a solvent to help dissolve them to aid in uniform distribution, and in some cases a thickening agent was added to the dough mixture to help finalise it for drying. The benefits, therefore, of the individualised jellies is that dosage can be customised to the weight of the rat without having to make a larger batch, thereby reducing wastage and being more cost-effective: the gelatine mixture is pipetted on top of the pre-weighed drug, which can remain in crystalline form. Furthermore, multiple rats can be habituated to/dosed with the jellies simultaneously, without the need for an experimenter to devote time to individually training/administering to the rats, as in the syringe-feeding method. As with other palatable oral methods, any aversive taste the drug may have should be masked by the palatable flavour of the jelly.

In conclusion, the current data demonstrate the efficacy of a jelly tablet as a reduced-stress alternative to oral gavage, as indexed by a pharmacokinetic profile for modafinil comparable to previous data from gavage administration, and an LMA profile comparable to previous i.p. administration of modafinil. Reducing stress-related arousal during the administration of pharmacological agents is a fundamental refinement to drug administration—and should be a goal for ethical experimentation regardless of any benefit to the data. Thus, whilst this technique should hopefully remove unwanted behavioural consequences that may mask the effects of a drug, it also promotes an overall ethical responsibility of reducing pain and distress in experimental animals.

## References

[CR1] Bastuji H, Jouvet M (1988). Successful treatment of idiopathic hypersomnia and narcolepsy with modafinil. Prog Neuro-Psychopharmacol Biol Psychiatry.

[CR2] Beracochea D, Celerier A, Borde N, Valleau M, Peres M, Pierard C (2002). Improvement of learning processes following chronic systemic administration of modafinil in mice. Pharmacol Biochem Behav.

[CR3] Bonnichsen M, Dragsted N, Hansen AK (2005). The welfare impact of gavaging laboratory rats. Anim Welf.

[CR4] Bowman EE, Xia S, Tait DS, Brown VJ (2014) Demonstrating a stress-free way to administer drugs during behavioural testing: modafinil restores attentional deficits in rats with lesions of the subthalamic nucleus. Paper presented at the Society for Neuroscience, Washington, DC,

[CR5] Brown AP, Dinger N, Levine BS (2000). Stress produced by gavage administration in the rat. Contemp Top Lab Anim Sci.

[CR6] Deroche-Gamonet V, Darnaudery M, Bruins-Slot L, Piat F, Le Moal M, Piazza PV (2002). Study of the addictive potential of modafinil in naive and cocaine-experienced rats. Psychopharmacology.

[CR7] Duteil J, Rambert FA, Pessonnier J, Hermant JF, Gombert R, Assous E (1990). Central α1-adrenergic stimulation in relation to the behaviour stimulating effect of modafinil; studies with experimental animals. Eur J Pharmacol.

[CR8] Edgar DM, Seidel WF (1997). Modafinil induces wakefulness without intensifying motor activity or subsequent rebound hypersomnolence in the rat. J Pharmacol Exp Ther.

[CR9] Ishizuka T, Murakami M, Yamatodani A (2008). Involvement of central histaminergic systems in modafinil-induced but not methylphenidate-induced increases in locomotor activity in rats. Eur J Pharmacol.

[CR10] Koob GF, Bloom FE (1988). Cellular and molecular mechanisms of drug dependence. Science.

[CR11] Leith NJ, Barrett RJ (1976). Amphetamine and the reward system: evidence for tolerance and post-drug depression. Psychopharmacologia.

[CR12] Mar AC, Nilsson SRO, Gamallo-Lana B, Lei M, Dourado T, Alsio J, Saksida LM, Bussey TJ, Robbins TW (2017). MAM-E17 rat model impairments on a novel continuous performance task: effects of potential cognitive enhancing drugs. Psychopharmacology.

[CR13] Minzenberg MJ, Carter CS (2008). Modafinil: a review of neurochemical actions and effects on cognition. Neuropsychopharmacology.

[CR14] Morgan RE, Crowley JM, Smith RH, LaRoche RB, Dopheide MM (2007). Modafinil improves attention, inhibitory control, and reaction time in healthy, middle-aged rats. Pharmacol Biochem Behav.

[CR15] Murphy HM, Ekstrand D, Tarchick M, Wideman CH (2015). Modafinil as a cognitive enhancer of spatial working memory in rats. Physiol Behav.

[CR16] Randall DC, Shneerson JM, File SE (2005). Cognitive effects of modafinil in student volunteers may depend on IQ. Pharmacol Biochem Behav.

[CR17] Roberts RA, Soames AR, James NH, Gill JH, Wheeldon EB (1995). Dosing-induced stress causes hepatocyte apoptosis in rats primed by the rodent nongenotoxic hepatocarcinogen cyproterone acetate. Toxicol Appl Pharmacol.

[CR18] Robinson ES (2012). Blockade of noradrenaline re-uptake sites improves accuracy and impulse control in rats performing a five-choice serial reaction time tasks. Psychopharmacology.

[CR19] Rowley HL, Kulkarni RS, Gosden J, Brammer RJ, Hackett D, Heal DJ (2014). Differences in the neurochemical and behavioural profiles of lisdexamfetamine methylphenidate and modafinil revealed by simultaneous dual-probe microdialysis and locomotor activity measurements in freely-moving rats. J Psychopharmacol.

[CR20] Simon P, Hemet C, Costentin J (1996). Analysis of stimulant locomotor effects of modafinil in various strains of mice and rats. Fundam Clin Pharmacol.

[CR21] Touret M, Sallanon-Moulin M, Jouvet M (1995). Awakening properties of modafinil without paradoxical sleep rebound: comparative study with amphetamine in the rat. Neurosci Lett.

[CR22] Turner DC, Robbins TW, Clark L, Aron AR, Dowson J, Sahakian BJ (2003). Cognitive enhancing effects of modafinil in healthy volunteers. Psychopharmacology.

[CR23] Walker MK, Boberg JR, Walsh MT, Wolf V, Trujillo A, Duke MS, Palme R, Felton LA (2012). A less stressful alternative to oral gavage for pharmacological and toxicological studies in mice. Toxicol Appl Pharmacol.

[CR24] Waters KA, Burnham KE, O'Connor D, Dawson GR, Dias R (2005). Assessment of modafinil on attentional processes in a five-choice serial reaction time test in the rat. J Psychopharmacol.

